# Primary care staff’s views and experience of patients’ online access to their electronic health record: a qualitative exploration

**DOI:** 10.3399/BJGP.2022.0436

**Published:** 2023-04-18

**Authors:** Gail Davidge, Lindsey Brown, Moira Lyons, Charlotte Blease, David French, Tjeerd van Staa, Brian McMillan

**Affiliations:** Centre for Primary Care and Health Services Research, University of Manchester, Manchester, UK.; Centre for Primary Care and Health Services Research, University of Manchester, Manchester, UK.; Centre for Primary Care and Health Services Research, University of Manchester, Manchester, UK.; Division of General Medicine, Beth Israel Deaconess Medical Center, Harvard Medical School, Boston, MA, US.; Manchester Centre of Health Psychology, University of Manchester, Manchester, UK.; Division of Informatics, Imaging & Data Sciences, University of Manchester, Manchester, UK.; Centre for Primary Care and Health Services Research, University of Manchester, Manchester, UK.

**Keywords:** digital health, electronic health records, primary health care, qualitative research, PAEHR, patient records access

## Abstract

**Background:**

NHS England have announced plans to enable all adult patients to have full prospective access to their primary care record by default. Despite this, little is known about the views and experiences of primary care staff regarding patients’ online records access (ORA).

**Aim:**

To examine the views and experiences of primary care staff regarding patients having online access to their primary care health record, and how this service could be supported and improved.

**Design and setting:**

A qualitative study of a purposive sample of 30 primary care staff in England.

**Method:**

Online semi-structured interviews with primary care staff were conducted between December 2021 and March 2022. Verbatim transcripts were analysed inductively using thematic analysis.

**Results:**

Most staff agreed with the principle of patient access to online health records but had mixed feelings regarding the potential benefits and drawbacks of applying this in practice. Staff identified opportunities for improving patient engagement, health literacy, and efficiencies in some administrative workloads, as well as concerns about maintaining the clinical integrity of patient records and ensuring that staff and patient safety and wellbeing are protected.

**Conclusion:**

Participants acknowledged that ORA may transform the purpose and function of the record and that ORA has potential to instigate a significant cultural shift in primary care, changing how staff work and relate to patients. This underlines the need for additional staff training and support to expand capability and capacity to adapt practice and enhance patient engagement with, and understanding of, their health records.

## INTRODUCTION

Recent systematic reviews on patient access to electronic health records highlight important outcomes in terms of improving patient engagement, safety, care, and clinical measures.^1^^–^3 Alongside this, UK Government policy on online records access (ORA) has evolved from the Access to Health Records Act 1990 to the National Information Board Framework, which stated that by 2018, *‘all citizens will have online access to their GP records and will be able to view copies of that data through apps and digital platforms of their choice’*.^4^ Further expansion of ORA continues to be firmly embedded in health and social care policy as part of the wider digital transformation agenda.^5^^–^7 This agenda is motivated by a commitment to enhance citizens’ access to health information with the aim of enabling patients to *‘become partners in managing their health’*.^8^

Since April 2020, the GP contract in England committed practices to offer patients online access to their primary care record.^9^ In November 2022, 48.8% of patients had signed up for at least one online service, but only 14.2% were able to view their detailed coded record (DCR).^10^ The DCR contains most information held in the record; however, full online access also includes free-text consultation notes and documents such as secondary care letters. Data regarding the percentage of patients with full records access is not publicly available, but the level of access granted varies, and low levels of access are a source of patient frustration.^11^

In response, NHS England (NHSE) announced a policy initiative to address inconsistencies in levels of patient online access to primary care records. This aims to ensure that almost all adult patients in England will be able to access all new prospective data (including free-text consultation entries) within their primary care health record via the NHS App or other online services by default.^8^ This announcement also includes confirmation of a longer-term ambition to enable patients to request historic coded records through the NHS App.^8^ Alongside these proposed changes, primary care has become increasingly reliant on remote access as a result of the coronavirus pandemic.^12^ Despite the potentially significant impact of such changes on how primary care staff practise, relatively little is known about the views and experiences of primary care staff in England, and what support might be required to ensure ORA is employed consistently and effectively.

**Table:** How this fits in

Previous research has noted primary care staff concerns about patients having online access to their health record, relating to issues such as: workload, safeguarding, patient confusion or distress, and health inequities. This study provides additional insights in the aftermath of the COVID-19 pandemic and in light of NHS England’s plans to enable full prospective records access for patients by default. Findings highlight that most primary care staff agree with patient records access in principle, and can see its potential benefits, but remain concerned about the impact on patient-centred care, safeguarding, and how to navigate this change. This study underlines the need for additional training and support for primary care staff to adapt their practice so they can address the needs of patients and protect patient safety and wellbeing while maintaining the clinical integrity of health records.

One survey, conducted over 10 years ago, reported positive views overall from English primary care staff with regards to ORA’s impact on workload, communication, trust, and patient self-management.^13^ Concerns that were identified related to time required to check records for third-party references, data protection issues, and practicalities, including forgotten passwords or hardware problems. After implementation, 20% of the clinicians surveyed reported changing the way they wrote notes and 73% said they would recommend the practice.^13^

In 2019, Louch *et al* explored the views of 19 primary care staff employed in a variety of clinical and non-clinical roles:^14^ responders were broadly supportive of ORA but reported uncertainty about the scope of access, types of information available, impact on relationships, and concerns about secure access and safeguarding. Another study conducted in 2019/2020, before the NHSE announcement, interviewed 16 general practice staff in England with experience of ORA. Findings highlighted concerns that ORA have a negative impact on the quality of record entries, patient safety, and workload.^15^ Combined, this research echoes findings from Sweden, the US, and Ireland.^16^^–^23

Given the recent developments aimed at accelerating patient records access, and in the context of an increasing reliance on digital tools resulting from the coronavirus pandemic, this present study aimed to explore primary care staff experiences of ORA in England, and to help further understanding of their views on the imminent changes. Specifically, the aims were to:

- explore the views and experiences of primary care staff regarding patients having online access to their electronic primary care health record; and
- examine what support primary care staff feel might be beneficial to maximise the benefits and minimise potential harms of ORA.

## METHOD

NHS Health Research Authority approval was applied for through the Integrated Research Application System online form and ethical approval was granted by the NHS Health Research Authority in December 2021. A purposive sampling strategy was employed to recruit primary care staff from a variety of roles, in a mix of rural and urban practices, with differing levels of records access, across the spectrum of socioeconomic deprivation.^24^ Recruitment methods included using the National Institute for Health and Care Research Clinical Research Networks, approaching colleagues, and snowballing recruits via recommendations from existing participants. A participant information sheet described the study in detail (see Supplementary Information S1). Interviews were conducted between December 2021 and March 2022. All participants gave written informed consent and data collection continued until data saturation was reached, whereupon no new themes were identified.^25^

### Semi-structured interviews

The interview topic guide (see Supplementary Information S2) was developed after extensive participatory and observational work, including attendance at NHSE awareness sessions, a workshop with 50 GP trainees, and in-depth discussions with primary care colleagues. The topic guide was also informed by reference to the literature, feedback from a patient and public involvement exercise, and discussions with an expert steering group. Participants were asked three closed-ended questions at the beginning of each interview. The first concerned what level of records access was granted to patients who request it at the practice where they worked. Participants were also asked to indicate level of agreement towards patients having online access to their primary care health record, including any free-text notes on a) an historic or b) a prospective, basis (Table 1). Interviews lasting 30–60 min were digitally audiorecorded and transcribed by a university approved service. Participants were recompensed with a £25 voucher.

**Table 1.** Participants’ (*N* = 30) responses to the closed-ended questions on records access

**Records access**	***n* (%)**
**Patients should have full historic records access**	
Agree	6 (20.0)
Somewhat agree	13 (43.3)
Disagree	3 (10.0)
Somewhat disagree	6 (20.0)
Don’t know	2 (6.7)

**Patients should have prospective records access**	
Agree	13 (43.3)
Somewhat agree	9 (30.0)
Disagree	2 (6.7)
Somewhat disagree	2 (6.7)
Don’t know	4 (13.3)

**Level of access currently granted to patients who request it^a^**	
None	0 (0.0)
Appointment booking/repeat scripts	6 (20.0)
Coded record	11 (36.7)
Full record including free-text entries	8 (26.7)
Don’t know/no answer	5 (16.7)

a *Practices,* n *= 24. When patients requested access, 5 (20.8%) practices granted appointment booking/repeat scripts, 11 (45.8%) granted access to the coded record, and 6 (25.0%) granted access to the full record. Data were unavailable for 2 (8.3%) of practices.*

### Demographics

Demographic details were collected at the beginning of each interview (see Supplementary Information S3). Twenty females and 10 males, aged between 25 and 64 years (mean 44.8 years, standard deviation 11.7) participated, from a range of primary care roles (Table 2).

**Table 2.** Participant characteristics (*N* = 30)

**Characteristic**	***n* (%)**
GP	9 (30.0)
Nurse	7 (23.3)
Receptionist	3 (10.0)
Practice manager	3 (10.0)
Healthcare assistant	2 (6.7)
Admin/secretarial	2 (6.7)
GP trainee	1 (3.3)
Physician associate	1 (3.3)
Pharmacist	1 (3.3)
Physiotherapist	1 (3.3)

**Education level**	
Postgraduate (level 7–8)	15 (50.0)
Degree (level 6)	9 (30.0)
Level 3–5	6 (20.0)

**Ethnicity**	
White	27 (90.0)
Asian	3 (10.0)

**Practice Index of Multiple Deprivation**	
Deciles 1–3 (most deprived)	13 (43.3)
Deciles 4–7	10 (33.3)
Deciles 8–10 (least deprived)	7 (23.3)

**Practice rural–urban classification**	
Urban	14 (46.7)
Suburban	10 (33.3)
Rural	6 (20.0)

### Analysis

An inductive thematic analysis approach^25^ was employed. Verbatim transcripts were analysed by two members of the research team using NVivo (version 12) software. The authors adopted a stepped approach, moving from initial and second readings of transcripts to familiarise themselves with the text before moving on to coding. Initial codes were generated and further refined, then themes were organised iteratively as the authors moved through the process until four key themes and corresponding subthemes were identified and the final analysis was agreed on by both researchers.

## RESULTS

### Qualitative themes

**Figure 1.**
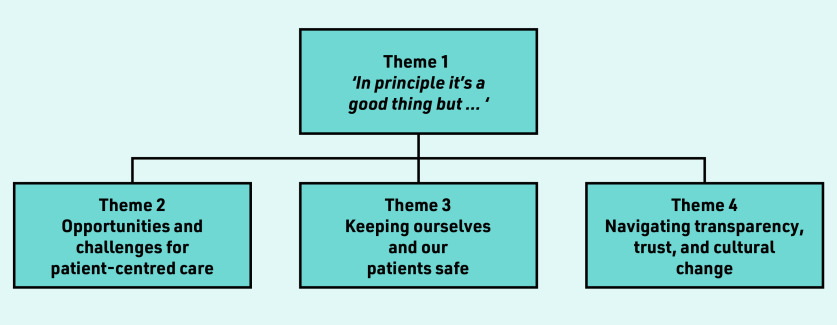
*Qualitative themes arising from primary care staff’s views and experiences of patient online records access.*

Primary care staff views and experiences of ORA concerned four main themes (Figure 1). The overarching theme of, *‘In principle it’s a good thing but … ’* encapsulated most responses. Almost all participants agreed with the idea in principle, but when applied to practice, conveyed a range of mixed feelings and concerns regarding trade-offs between potential benefits and risks, which are summarised within the remaining three themes.

### Theme 1: *‘In principle it’s a good thing but … ’*

Participants considered ORA to be a step forward in acknowledging patients’ existing rights, aligning with formal legal and contractual responsibilities:^9^

*‘It’s their health, they deal with it 365 days a year, they should be able to have access to it.’*

(Participant [P]02, GP, practice grants access to coded record)

Those with experience of ORA stated that many of their initial fears had not been realised, while others acknowledged resistance among colleagues, but thought that this:

*‘... will go once it happens and the world doesn’t end.’*

(P04, GP, practice grants access to coded record)

A third of participants conveyed scepticism about the rationale for accelerating ORA:

*‘I’m not anti it as a fundamental concept … but it feels it’s been done too quickly and without the right people involved and without thinking … and for the minimal benefits that we are likely to get, the risks are huge.’*

(P28, GP trainee, practice grants access to the full record)

Although others agreed in principle, they wanted more evidence and expressed disappointment regarding the level of consultation with clinicians regarding planned changes. Some staff considered that the use of existing electronic communication software, such as ‘accurx’ (https://www.accurx.com), which enables two-way communication between health professionals and patients via SMS messaging, was an adequate alternative to enabling patients’ online access to their full record. Others considered existing provision of making a ‘subject access request’ was sufficient.

A small proportion of participants were resigned that, regardless of personal views, readjustment was inevitable:

*‘It’s coming, whether we like it or not, so it’s just getting ready for it really.’*

(P25, GP, practice grants access to coded record)

### Theme 2: Opportunities and challenges for patient-centred care

#### Patient ownership, empowerment, and control

Access to free-text entries encouraging patients to take more responsibility for their healthcare needs was mentioned frequently:

*‘If you don’t give them access to their medical records, then it’s difficult for them to recognise or accept that responsibility.’*

(P19, GP, practice grants access to coded record)

Over two-thirds of participants identified opportunities for greater patient autonomy, and this was seen as mutually beneficial to patients and staff. For example, sharing records with healthcare professionals away from home, self-referrals to private providers, checking immunisations, or locating information for health insurance or benefit applications. Others pointed out other advantages such as patient engagement with longer-term health management.

Staff also considered that ORA could be used to demonstrate clinician accountability, and that increased transparency offered patients reassurance that:

*‘Nothing’s being kept from them.’*

(P14, nurse, practice grants access to the full record)

#### Patient activation and health literacy

Despite the fact that concerns were often raised about patients’ capacity to understand the content of their records, ORA was also seen as a convenient gateway to improve patients’ health management and literacy. For example, supporting patients to develop greater health awareness and requisite skills to monitor progress towards individual goals and adopt positive lifestyle changes:

*‘The patient may see that risk factor and decide to do something about it.’*

(P10, nurse, practice grants access to appointment booking/repeat scripts)

#### Confirmation and reassurance

Staff recognised that patients do not always retain information about plans or diagnoses, and would benefit from opportunities to digest this information outside the consultation. Offering patients reassurance that their concerns have been taken seriously or providing confirmation that follow-on tests or secondary care appointments have been actioned were also valued:

*‘… they can check back and be like, okay, you said that we would see each other in 8 weeks’ time, so that’s the plan, so I feel secure that she’s not forgotten about me.’*

(P25, GP, practice grants access to coded record)

#### Communication, integration, and involvement of others in patient-centred care

ORA was perceived as a tool for improving communication and involvement with carers as well as coordinating information across other NHS services, offering greater informational continuity, organisational efficiency, and joined up patient care across community and secondary care services:

*‘I found it particularly useful for acting as a proxy for an older relative … It’s that enabling family to be more involved, with the patients’ consent and help, it’s absolutely invaluable.’*

(P06, clinical data manager, practice grants access to full record)

#### Equity of access for all

Potential to further entrench the digital divide and widen health inequalities was raised as a significant problem:

*‘Around here, like I say, there’s a lot of poverty, not everybody has a mobile phone, not everybody has a computer. Not everybody has the ability or affordability to borrow things or even get buses into town into libraries, and stuff.’*

(P12, healthcare assistant, practice grants access to appointment booking/repeat scripts)

Concerns were raised about disadvantaging patients with sensory needs, learning disabilities, low levels of literacy, or for whom English is not their first language. Additional risks for patients reliant on family members, third-parties, or computer-generated translation services were also highlighted. Despite this, some considered that ORA could remove barriers for patients who experience difficulties communicating verbally or face to face.

### Theme 3: Keeping ourselves and our patients safe

#### ORA may make patients feel worse or put their safety at risk

A large proportion of concerns centred on the potential for patients to experience greater anxiety, distress, or offence because of not fully understanding information in their record. Other participants considered patients best placed to decide whether or not they wish to view their records:

*‘It’s about balance and giving your patients some credit and autonomy and not assuming that they’re all idiots and that they can cope with truthful information.’*

(P05, receptionist, practice grants access to full record)

Notes containing clinicians’ thought processes were considered important in terms of safety and continuity of care. Significant concerns were raised regarding conflicts of interest around maintaining the clinical integrity of notes, protecting patients’ wellbeing, and managing medicolegal requirements:

*‘… the 17-year-old that’s rung up to tell me they’re going to kill themselves, and I need to write notes that explain why I haven’t sent the police around … they are protecting me a little bit as well … I think if you were in a bad place … reading someone’s clinical interpretation, clinical and slightly legal worry-avoiding notes … could be pretty blooming upsetting.’*

(P28, GP trainee, practice grants access to full record)

#### Safeguarding and third-party access

Unauthorised access to content that may jeopardise patient safety was a frequent source of concern:

*‘What if I forget to do that* [redact information]*, what if something goes wrong with that? There could be absolutely disastrous consequences in that situation.’*

(P29, GP partner, practice grants access to coded record)

Uncertainty about patient capacity or authorising proxy access for parents, families that included a minor as the only English speaker, or spouses with access to notes containing sensitive details (for example, termination of pregnancy) were highlighted as potentially contentious. Participants expressed significant concerns about patients at risk of domestic violence or children and older people being placed at a greater risk of being coerced into sharing their record online and perceived this as an issue that is more difficult to control outside of the safety of a physical consultation. These concerns were raised by participants, regardless of the level of online access their practice currently offered, as potential risks, rather than being based on direct experience.

#### ORA can help improve patient safety and continuity of care

Benefits regarding the quality of clinical data, patient safety, and continuity of care were identified by staff. For example, patients updating incorrect contact details or diagnoses, and accessing contemporaneous clinical information away from home. Participants acknowledged that patient records are likely to include errors and expressed apprehension about managing patient enquiries and correcting content. Practices with experience of ORA gave examples of encouraging patients to check their record accuracy and perceived this as an opportunity to improve safety:

*‘If you encourage them to say, look, if something’s not right, tell us and we’ll look at it together, I think that’s the best way forward.’*

(P06, clinical data manager, practice grants access to full record)

#### Staff wellbeing and workload burdens

Participants suggested that reductions in administrative tasks may be outweighed by increased patient enquiries or more time spent writing notes. Anxieties about the impact of greater transparency on staff wellbeing were raised in terms of exposure to increased cognitive burden and risk of litigation. Others considered that impact on workload would be minimal but acknowledged that adaptations to workflow may be required:

*‘If anything, workload will just change.’*

(P17, reception manager, practice grants access to appointment booking/repeat scripts)

### Theme 4: Navigating transparency, trust, and cultural change

#### Navigating cultural shifts: this will change how we do our job

Participants recognised ORA has potential to build or undermine trust. Movement away from paternalistic interaction and dismantling of ‘hierarchies’ was highlighted as a benefit of greater transparency as both parties have access to the same level of information and the consultation note *‘really becomes a shared document’* (P19, GP, practice grants access to the coded record).

Clinicians recognised their duty of candour, and some perceived ORA as a necessary reflection of this. However, it was acknowledged that candour can sometimes be problematic for maintaining positive relationships. Nonetheless, staff noted opportunities for building trusting relationships and reassurance by demonstrating acknowledgement of patients’ concerns:

*‘You read it and think … she really listened and cared, she’s taking onboard stuff … I feel quite comforted.’*

(P05, receptionist, referring to experience of viewing their own consultation note online)

Concerns were raised about patients disagreeing with record content and this undermining trust and damaging relations. Staff also raised concerns about reputational risks or that they may write less because of fears that records could be published on social media:

*‘I think some people are quite aware or afraid of writing something that will be misunderstood or … can be screen grabbed off a mobile device and put on Facebook … that’s then in the public domain then and they’ve* [staff] *got no control of where it goes. I know some people, we’ve talked about it in the staff room, and some people are quite cautious about what they write to the point where they write less.’*

(P23, nurse associate, practice grants access to full record)

Participants lamented the loss of a ‘safe space’ to record details that may help to build and nurture relationships with patients:

*‘It’s those little human details that help you remember that it’s Jenny in front of you, not the abdo* [abdominal] *pain.’*

(P28, GP trainee, practice grants access to full record)

Staff acknowledged that ORA may help ensure prospective records address patients with greater compassion and respect:

*‘… that’s going to have to be a bit of a cultural change for all of us … we might have to rethink our language, especially around things like drug seeking behaviour or concerns about safeguarding.’*

(P25, GP, practice grants access to coded record)

#### Patient online access changes the function and purpose of the health record

Greater transparency was considered to constitute a fundamental shift in the purpose and function of the record that necessitates transformations to how staff and patients communicate and relate to one another. Many participants declared that they do not currently write notes with a patient audience in mind. GPs acknowledged that ORA may motivate clinicians to think more carefully about the content and style of notes. Some clinicians recognised that adaptations to documentation could transform the quality of notes with clearer plans for patients to follow and ultimately improve patient health literacy and engagement.

However, there was also some debate about whose interests medical notes primarily serve and concerns raised about how to ensure that both clinicians and patients’ needs are addressed. Concerns were also raised about increased cognitive burden and workload adapting notes for patient understanding. Clinicians expressed hesitance around describing a patient’s presentation and gave examples of feeling wary about recording details such as ‘patient smelled of cannabis’ or ‘obese’ due to fears that this may damage relationships:

*‘… we’ll fundamentally change how and why we’re recording notes. I think it’ll make us less safe because I won’t be writing “query Ca* [cancer]*”. And then the next person might not think about that or I won’t think about it again or I’ll think that’s been ruled out. Or I can’t write a clear set of notes that explains my thinking and what I’m doing because it will upset the patient.’*

(P28, GP trainee, practice grants access to full record)

#### ‘We need help and support to get ready for this’ — resources, training, and support needs

Participants also highlighted that continuity and consistency would be difficult to maintain because of a wide variation of approaches:

*‘Some people are really good at it and emotionally intelligent and some people are pretty crap at it … You only need to read a section of your colleague’s notes to realise that we’re all very different in how we document things.’*

(P20, nurse, practice grants access to coded record)

Concerns were raised about non-clinical staff managing patient enquiries regarding clinical content. Staff considered that additional training and resources, including dedicated personnel to manage ORA issues, would be essential for larger practices. Staff from practices already offering ORA commented that additional clinical data management roles had been especially useful. The following quote refers to dedicated drop-in clinics, which have been set up to support patients with records access queries:

*‘… there’s no fear now if someone rings up about a records access issue, they just pop it into this clinic for the person who is experienced to sort it.’*

(P15, practice manager, practice grants access to full record)

Few staff had access to their own health record and considered that experience of this would help to gain a better understanding of a patient’s perspective. In particular, staff requested specialised training on dealing with patient queries and complaints, redaction and safeguarding, proxy access, ascertaining capacity, medicolegal considerations, and data protection. Uncertainty around how to write more effectively for patient understanding while maintaining the clinical integrity of patients’ records was raised as a significant concern.

## DISCUSSION

### Summary

Very few participants disagreed with ORA in principle, although views differed with respect to optimism, ambivalence, or resignation about forthcoming changes. The impact of increased transparency was raised as a key issue that could enhance patients’ capacity to be more informed and involved in decisions about their care alongside an acknowledgement that this may also change the nature of their relationships with staff. Participants acknowledged that ORA may transform the purpose and function of the record and that ORA has potential to instigate a significant cultural shift in primary care, changing how staff work and relate to patients. This underlines the need for additional staff training and support to expand capability and capacity to adapt practice and enhance patient engagement with, and understanding of, their health records.

### Strengths and limitations

This study was undertaken after NHSE announced plans to accelerate citizen access to GP records. It offers timely insights about the views of a wide range of primary care staff on anticipated challenges and opportunities that surround putting policy into practice. Including participants from practices that already elected to offer patients access to their full online record, as well as practices with no prior experience, adds further weight to this study. The diversity of the sample in terms of range of roles, responsibilities, settings, and socioeconomic characteristics are a further strength. Eliciting views through one-to-one interviews enabled participants to express their thoughts and share examples of existing practice without fear of reproach from colleagues. This approach facilitated further in-depth understanding of views and experiences; however, the methodology limited opportunities for wider group discussion between different members of staff to consider how practices might usefully adapt to forthcoming changes as a team.

### Comparison with existing literature

This study, to the authors’ knowledge, is unique in that it reflects the views of primary care staff in England on the cusp of a significant policy change and during the aftermath of a global pandemic. The findings are congruent with earlier studies undertaken before the NHSE announcement, and before the coronavirus pandemic.^14^^,^^15^ This study adds further depth and insight regarding staff recognition of the capacity of ORA to expand and promote patient autonomy, as well as offering scope to diminish paternalistic attitudes. As in this study, previous research reported that staff broadly agreed with ORA in principle, and recognised benefits in terms of patient safety, improving patient engagement, health literacy, and workload efficiencies. Similarly, in a US survey of clinicians with several years’ experience offering patients access to their free-text entries, more than two-thirds reported that they supported ORA.^21^

Concerns about the potential for increased workload, patient enquiries, and complaints have previously been reported;^14^^,^^15^ fears echoed by staff anticipating forthcoming policy change within this study. Research exploring the impact of ORA in the US has yielded mixed results. One year-long intervention providing patients with electronic links to their primary care notes found very few doctors reported longer consultations or more time addressing patients’ questions outside of consultations,^26^ but another found that, after implementation of ORA, there was a doubling in the number of messages sent by patients within the 6 h after patients reviewed a result.^27^

Anxieties about increased transparency and maintaining the clinical function of patients’ notes as a consequence of ORA are underlined in previous work.^14^^,^^15^ This study confirms uncertainty persists around recording thought processes or safeguarding information in a manner that is not detrimental to patient wellbeing, safety, or continuity of care, and adds specific empirical examples of clinicians’ key concerns in this regard.

In line with results of a previous survey,^28^ changes in documentation practices as a result of ORA has the potential to influence the quality of patient care in both positive and negative ways. The current study found that staff were concerned about potential increases in workload and cognitive burden but also perceived some benefits in that ORA may improve record clarity and content. The participants in the current study also pointed out opportunities to improve relationships and flatten traditional hierarchies by transforming patient notes into a shared care document.

On a positive note, studies suggest after accessing their records, the most disadvantaged patient populations report more benefits than other patients.^29^^,^^30^ However, this study reinforces previous research findings on the need to support equitable records access for patients,^14^^,^^15^ and this issue remains a significant concern. For example, recent studies have explored objective linguistic features of documentation and found stigmatising language tends to be more common in free-text entries written about some already marginalised patient populations.^31^^–^33 There is a need to ensure that documentation practices deliver content that is objective, factual, and written in a style that patients can understand.

This study offers additional insights regarding the necessity for staff support and training, and the importance of staff confidence and preparedness to adapt their practices to meet the needs of patients accessing their record while continuing to maintain their clinical function and protect patient safety and wellbeing. These aspects have not been reported in detail in previous work.

### Implications for research and practice

Accelerating ORA in primary care is a key aspect of realising wider digital transformation policy ambitions set out in the UK Government’s current plans for digital health and social care.^34^ This study has highlighted that staff have mixed feelings and significant concerns about putting this policy into practice. This underscores the importance of ensuring all staff are informed and well supported to adapt working practices, for example, through provision of training on how to make free text more accessible to a lay audience,^35^ or when redaction may be justified.^8^ Although most staff agreed with the principles behind ORA, many are yet to be convinced about managing this alongside existing workloads or that the benefits will outweigh perceived risks. These concerns need to be addressed, alongside ensuring that practices can access resources and training required to provide online access safely, effectively, and equitably. Subsequent evaluation of primary care staff and patients’ experiences of the realities of ‘putting principles into practice’ is an important topic for future research.
